# Association Between Statin Use, Cytokine Storm, and Clinical Outcomes in COVID-19 Patients with Diabetes

**DOI:** 10.3390/biomedicines14030518

**Published:** 2026-02-26

**Authors:** Ivan Feldi, Barbara Grubišić, Tatjana Bačun, Zvonimir Bosnić, Dunja Šojat, Mario Šafer, Marko Pirić, Lada Zibar

**Affiliations:** 1 Faculty of Medicine Osijek, Josip Juraj Strossmayer University of Osijek, Josipa Huttlera 4, 31000 Osijek, Croatia; ivanfeldi@gmail.com (I.F.);; 2 Department of Internal Medicine, General County Hospital Našice, Ul. bana Jelačića 10, 31500 Našice, Croatia; 3 Department of Infectious Diseases, University Hospital Centre Osijek, Josipa Huttlera 4, 31000 Osijek, Croatia; 4 Division of Endocrinology, Department of Internal Medicine, University Hospital Centre Osijek, Josipa Huttlera 4, 31000 Osijek, Croatia; 5 Department of Clinical Medicine, Faculty of Dental Medicine and Health Osijek, Josip Juraj Strossmayer University of Osijek, Crkvena Street 21, 31000 Osijek, Croatia; 6 Health Centre of Požega-Slavonija County, Ulica Matije Gupca 10, 34000 Požega, Croatia; 7 Health Centre of Osijek-Baranja County, Park Kralja Petra Krešimira IV. 6, 31000 Osijek, Croatia; 8 Department of Internal Medicine, General Hospital Virovitica, Lj. Gaja 21, 33000 Virovitica, Croatia; 9 Department for Nephrology, Internal Clinic University Hospital Merkur, Zajčeva 19, 10000 Zagreb, Croatia

**Keywords:** COVID-19, diabetes mellitus, hydroxymethylglutaryl-CoA reductase inhibitors, cytokine release syndrome, interleukin-6, ferritins

## Abstract

**Objective**: To examine patterns of inflammatory markers, kidney function, and clinical outcomes in patients with type 2 diabetes (T2D) hospitalized for COVID-19, with particular focus on the impact of therapy with hydroxymethylglutaryl-CoA reductase inhibitors (statins). **Methods**: We conducted a retrospective analysis of 440 T2D patients, divided into those who were on statin therapy prior to hospitalization and those who were not. Clinical characteristics, laboratory markers of inflammation, kidney function, and the requirement for invasive ventilation were compared between the groups. **Results**: Patients were predominantly older adults with a high burden of comorbidities, most commonly arterial hypertension and chronic kidney disease, and presented with elevated inflammatory markers and hyperglycemia at admission. Statin users more frequently had cardiovascular comorbidities and showed lower unadjusted interleukin-6 (IL-6) and ferritin levels, although these differences were not significant after multivariable adjustment. Importantly, statin therapy was independently associated with lower odds of mechanical ventilation in multivariable analysis. **Conclusions**: Despite a higher prevalence of cardiovascular comorbidities, diabetic patients receiving statins had lower odds of mechanical ventilation. No independent association with inflammatory markers was confirmed, and the observed relationship with respiratory outcomes should be interpreted cautiously given the observational design.

## 1. Introduction

The Coronavirus disease-19 (COVID-19) pandemic, caused by severe acute respiratory syndrome coronavirus-2 (SARS-CoV-2), was the defining global health crisis during the period 2020–2023, affecting public health, social life, economic strategies and political efficacy. This global phenomenon was first identified in Wuhan, China, and quickly spread, affecting more than 770 million people worldwide [1,2].

The rapidly evolving pandemic enhanced the necessity of a better understanding of the molecular events involved in the interactions between the host and SARS-CoV-2, trying to shed light on developing improved therapeutic agents to selectively inhibit the hyperinflammation signaling pathway in order to reduce morbidity and mortality among affected patients [3].

Several studies have shown that most of the severe and critically ill COVID-19 patients had preexisting chronic comorbidities, including hypertension, type 2 diabetes (T2D), cardiovascular diseases (CVD), and respiratory diseases, which are known to perturb the levels of cytokines, chemokines, and angiotensin-converting enzyme 2 (ACE2) [4].

Further clinical evidence showed that patients with severe COVID-19 had elevated proinflammatory cytokines, such as interleukin-1 beta (IL-1β), interleukin-2 (IL-2), interleukin-6 (IL-6), interleukin-7 (IL-7), interleukin-8 (IL-8), tumor necrosis factor alpha (TNF-α), C-C motif chemokine ligand 2 (CCL2), macrophage inflammatory protein-1 alpha (MIP-1α), and C-X-C motif chemokine ligand 10 (CXCL10, also known as interferon gamma-induced protein 10 or IP-10). This surge in inflammatory molecules is commonly referred to as cytokine release syndrome or a cytokine storm in COVID-19 [5]. IL-6 is a key proinflammatory cytokine that plays an important role in the human immune response [6]. It is central to the development of the cytokine storm [7].

The pathophysiology of COVID-19 and diabetes mellitus is interlinked, and diabetes mellitus is associated with severe COVID-19 outcomes [8]. Also, COVID-19 can contribute to the worsening of diabetes mellitus and may also play a role in the development of new-onset diabetes [9].

The study by Tao and colleagues looked into acute diabetes complications in patients hospitalized with COVID-19 who already had diabetes [10]. They found that higher levels of procalcitonin (PCT) and C-reactive protein (CRP) were strong predictors of hyperglycemic crises like diabetic ketoacidosis (DKA) and hyperosmolar hyperglycemic state (HHS) in patients with T2D diabetes and COVID-19. Interestingly, though, these markers did not seem to predict the overall outcomes of the illness, including mortality.

These findings further support the increasing evidence that SARS-CoV-2 infection can act as a significant and clinically relevant trigger for diabetic ketoacidosis (DKA), particularly in individuals with preexisting diabetes [10]. Previous multicenter studies in Croatia had already identified infections and poor glycemic control as the most common triggers for DKA, but the new insights related to COVID-19 emphasize how important it is to recognize and actively manage these patients early on [11].

There is a bidirectional relationship between COVID-19 and diabetes. On the one hand, diabetic patients have a higher risk of developing complications when they present with COVID-19, and, on the other, SARS-CoV-2 may act as a diabetogenic agent by binding to ACE2 receptors on pancreatic beta cells, leading to acute dysfunction and altered glucose metabolism [12].

Interacting with other risk factors, hyperglycaemia might modulate immune and inflammatory responses, thus predisposing patients to severe COVID-19 and a possible lethal outcome. Potential pathogenetic links between COVID-19 and diabetes include effects on glucose homeostasis, inflammation, altered immune status and activation of the renin–angiotensin–aldosterone system (RAAS) [13].

**Figure 1:**
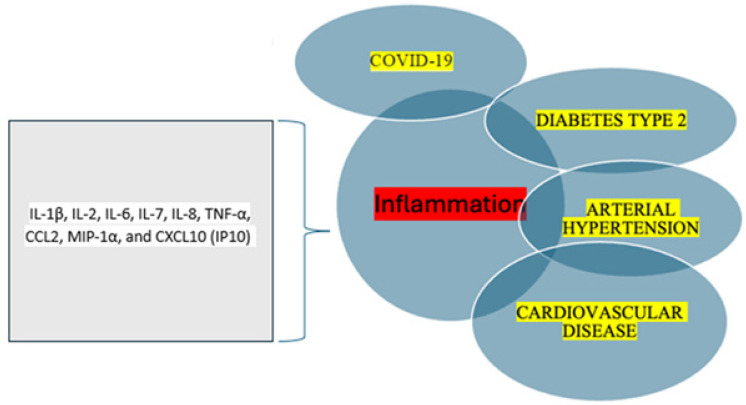
Bidirectional relationship between various chronic diseases and coronavirus disease-19 (COVID-19). IL-1β (interleukin-1 beta), IL-2 (interleukin-2), IL-6 (interleukin-6), IL-7 (interleukin-7), IL-8 (interleukin-8), TNF-α (tumor necrosis factor alpha), CCL2 (C-C motif chemokine ligand 2), MIP-1α (macrophage inflammatory protein-1 alpha), and CXCL10 (C-X-C motif chemokine ligand 10).

The complex bidirectional interactions between chronic diseases and COVID-19, including the role of key proinflammatory cytokines and chemokines, are illustrated in Figure 1.

To date, limited data exist on the impact of the COVID-19 pandemic on chronic complications in patients with diabetes. Optimizing metabolic management may help improve prognosis and reduce the burden on healthcare systems.

### 1.1. Role of Inflammation in the Pathogenesis of Cardiometabolic Disease

Inflammation plays a central role in the development and progression of chronic cardiometabolic diseases, including T2D, and serves as a key link between hyperglycemia and cardiovascular complications [14]. It is reflected by elevated circulating proinflammatory mediators such as IL-6 and CRP, which contribute to endothelial dysfunction, insulin resistance, and tissue damage, potentially worsening the clinical course and outcomes in patients with COVID-19 [15]. Clustering inflammatory biomarkers with clinical and sociodemographic characteristics has been shown to support clinical reasoning and reduce patient complexity in individuals with T2D [16].

### 1.2. Statins and Their Effects on T2D in SARS-CoV-2

Although the West of Scotland Coronary Prevention Study (WOSCOPS) demonstrated that pravastatin can reduce the risk of developing T2D, subsequent meta-analyses have reported an association between hydroxymethylglutaryl-CoA reductase inhibitors (statins) use and an increased risk of T2D [17,18]. A meta-analysis encompassing 14 randomized controlled trials with approximately 95,000 participants without T2D showed that statin use was associated with an approximately 11% relative increase in the risk of new onset T2D. Subgroup analyses indicated that atorvastatin and rosuvastatin were most strongly associated with incident T2D [18]. Despite this elevated risk, the cardioprotective benefits of statins outweigh the risk of developing T2D, as confirmed by current guidelines for the management of cardiovascular disease in patients with diabetes [19].

Statins are widely used lipid-lowering agents with well-established cardiovascular benefits. Emerging evidence suggests that, beyond lipid control, statins may exert anti-inflammatory effects that could influence outcomes in patients with infections, including COVID-19. In a study by Saeed et al. involving 4252 COVID-19 patients in New York, statin use was associated with lower inflammatory markers and reduced mortality in patients with diabetes, while no significant effect was observed in non-diabetic patients [20]. Similarly, a study in Detroit reported lower mortality among statin users [21]. These findings suggest that the anti-inflammatory and protective effects of statins may be more pronounced in patients with diabetes.

Statins may exert multiple beneficial effects in COVID-19. By lowering plasma lipid levels, they can alter the structure of lipid rafts in cell membranes, potentially hindering SARS-CoV-2 entry and impairing viral replication, which relies on lipids for membrane formation and replication complexes. Statins may also increase ACE2 expression, yet evidence suggests this does not enhance viral entry due to changes in lipid raft composition and receptor localization. Additionally, statins can inhibit viral proteins critical for replication, such as Mpro (Main protease) and RdRp (RNA-dependent RNA polymerase), modulate immune responses by reducing macrophage activation, improve endothelial function, and decrease thrombotic risk, all of which may mitigate severe COVID-19 complications [22,23].

T2D is a major public health concern, with cardiovascular disease being its most critical comorbidity. Personalized approaches to managing T2D in COVID-19 are emerging, focusing on anti-inflammatory strategies. While several drugs with anti-inflammatory properties may reduce mortality in diabetic patients, a better understanding of the mechanisms underlying cytokine storm in diabetes could guide future treatment strategies, improving early diagnosis and reducing morbidity and mortality [5].

This study aimed to explore the interplay between chronic inflammation in diabetic patients, acute COVID-19-induced immune dysregulation, and the potential modulatory effects of statins.

## 2. Materials and Methods

### 2.1. Participants and Study Design

Participants were patients diagnosed with T2D who were admitted to the University Hospital Centre Osijek (Osijek, Croatia) due to SARS-CoV-2 infection. They were divided into two groups according to statin use: those who were treated with statin therapy prehospitally, and those who were not, in order to determine how that was associated with inflammatory response and outcomes in diabetic COVID-19 patients.

**Figure 2:**
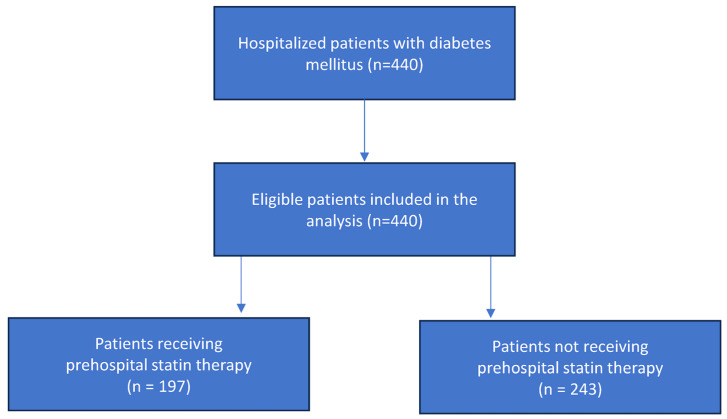
Flowchart of patient inclusion and grouping.

The study type was a historical cohort study. The data were collected between October 2020 and June 2021. The Expert and Ethics Council of the Faculty of medicine Osijek (Osijek, Croatia) approved the study (ID: 2158-61-07-21-167). Patient inclusion and grouping are shown in Figure 2.

The exclusion criteria were patients under 18 years of age, pregnant women, and those who had been vaccinated against SARS-CoV-2. Blood samples for laboratory analyses were collected at hospital admission.

### 2.2. Data Collection

Information on participants’ sociodemographic characteristics, anthropometric measurements, comorbidities, medications, specific diabetic treatments, length of hospital stay, and laboratory tests reflecting metabolic and renal status, as well as inflammation, was collected. Most of the data were obtained from patients’ medical records.

General obesity was assessed using body mass index (BMI, kg/m^2^). Renal function decline was evaluated from serum creatinine, patient age, and sex using the Chronic Kidney Disease Epidemiology Collaboration (CKD-EPI) equation. An estimated glomerular filtration rate (eGFR) ≤ 60 mL/min was considered indicative of decreased renal function, corresponding to CKD stages 3–5.

Other major diabetic complications, including cardiovascular diseases such as coronary artery disease (CAD), chronic heart disease (CHD), and cerebrovascular disease, were recorded from medical histories if confirmed by specialist examinations. Information on CHD severity was not always available because cardiac imaging data were not systematically recorded.

Data on prescribed medications, including main antidiabetic and antihypertensive drugs, statins, and nonsteroidal anti-inflammatory drugs (NSAIDs), were collected, as these could influence metabolic parameters or inflammation markers.

Ferritin and lactate dehydrogenase (LDH) were measured as indicators of tissue damage, along with classical markers such as C-reactive protein (CRP), white blood cell count, hemoglobin, and hematocrit. All laboratory parameters were analyzed upon admission to the hospital. IL-6, an important cytokine in the cytokine storm, was measured by separating plasma from part of the blood samples in heparinized tubes, followed by centrifugation. Samples were transported under refrigerated conditions to the Laboratory for Clinical Immunology and Allergology Diagnostics at the University Hospital Centre of Osijek.

Additionally, concentrations of IL-6 and PCT were measured in the same serum using an electrochemiluminescence immunoassay (ECLIA) on the COBAS e601 immunoassay analyzer (Roche Diagnostics GmbH, Mannheim, Germany). All laboratory parameters were determined during the hospital stay independently of this study, as part of routine diagnostic and clinical procedures in pandemia.

### 2.3. Statistical Analysis

Categorical variables were presented descriptively as absolute and relative frequencies, numerical variables as arithmetic mean and standard deviation or median and interquartile range, depending on the normality of the distribution. The normality of the distribution of variables was determined by the Kolmogorov–Smirnov test. For comparison of numerical variables, Student’s *t*-test (ANOVA for more than 2 samples) or the Mann–Whitney U test (Kruskal–Wallis test for more than 2 samples) was used, depending on the sample size and normality of the distribution, and for comparison of categorical variables, Fisher’s exact test or the Chi-square test was used. Additionally, multivariate logistic regression was used to assess factors associated with mechanical ventilation, and multivariate linear regression was used for log-transformed IL-6 and ferritin levels to account for non-normal distribution. A *p*-value < 0.05 was considered statistically significant.

## 3. Results

In the group of 440 examined diabetic patients, participants were mostly 60–77 years old, and men participated more than women (Table 1).

Clinical and anthropometric characteristics of the patients are presented in Table 2. With respect to comorbidities, arterial hypertension was the most common (in 87% of all patients. CKD was the second most common. Of non-cardiovascular comorbidities, the descriptive data indicated a small proportion of liver disease (Table 3). Most of the patients had been prescribed antihypertensive drugs of the ACE-INH/ARB (angiotensin-converting enzyme inhibitors/angiotensin receptor blockers) group.

According to the day of illness at admission, most patients had been symptomatic for seven days, with a median of 11 days (IQR 7–15) of duration of hospitalization (Table 1).

According to their lab results, most of the patients had elevated levels of acute inflammatory markers (measured by CRP and leukocytes), with a normal range of cytokine IL-6. Other markers of inflammation were also elevated (ferritin and LDH), with a normal range of thrombocytes (Table 4). With regard to clotting factor indicators, D dimers were elevated.

About one-third of participants had CKD. In most cases, renal function was only mildly reduced (eGFR 90–60 mL/min). Most patients had elevated blood glucose levels, consistent with the inflammatory state. LDL cholesterol levels showed a median value of 2.7 mmol/L in the study population (Table 4). IL-6 was available for 343/440 patients, ferritin for 329/440, PCT for 369/440, and CRP for 437/440 patients. Analyses were conducted on complete cases for each parameter.

A high proportion of patients had been prescribed antihypertensive drugs of the ACE-INH/ARB group and hypolipidemic statin drugs, as seen in Table 5. The traditional antidiabetic drug metformin was the most prescribed, while a moderate or low number of newer generation antidiabetic drugs, such as glucagon-like peptide-1 receptor agonists (GLP-1 RAs) and sodium-glucose cotransporter-2 (SGLT-2) inhibitors, were also used. The distribution of antidiabetic therapy is shown in Table 6.

Forty-five percent (n = 197) were on statin therapy. Most patients were on atorvastatin (75%), rosuvastatin (13%), or simvastatin (10%), while only 2% were on fluvastatin. Patients were also categorized according to statin potency. Among the 197 patients on statin therapy, 3 (1.5%) received low-potency, 133 (67.5%) received moderate-potency, and 61 (31%) received high-potency statins. The frequency, type of statin therapy and statin potency are presented in Table 7.

The whole population was divided into two groups: one on statin therapy and the other not on statin therapy. As shown in Table 8, patients on statin therapy significantly more often had arterial hypertension (94% vs. 80%, *p* < 0.001), coronary disease (38% vs. 13%, *p* < 0.001, heart failure (29% vs. 19%, *p* = 0.009), CKD (42% vs. 22%, *p* < 0.001), and cerebrovascular disease (33% vs. 15%, *p* < 0.001) (Table 8).

In the multivariate logistic regression model adjusted for predefined baseline confounders, statin therapy was independently associated with lower odds of mechanical ventilation (OR = 0.48, *p* = 0.03). Lower SpO_2_ at admission was also independently associated with mechanical ventilation (OR = 0.90, *p* < 0.001) (Table 9).

Median age was the same in the two groups (patients with statin 70 years (64–77) vs. patients not on statin therapy 70 years (62–78), *p* = 0.635; Mann–Whitney U test). There was a similar median length of stay in hospital in patients on statin therapy and not on statin therapy. Median heart rate was significantly slower in patients on statin therapy (88 (77–99) vs. 90 (80–102), *p* = 0.029, Mann–Whitney U test). Patients on statin therapy had significantly lower hemoglobin levels (129 (116–143) vs. 134 (122–146), *p* = 0.007, Mann–Whitney U test). In relation to inflammatory markers, there was no significant difference between the two groups in leukocyte number (6.9 (5.3–9.1) vs. 7.4 (5.1–9.9), *p* = 0.283, Mann–Whitney U test), CRP (99 (52–158) vs. 133 (66–166), *p* = 0.208, Mann–Whitney U test) and PCT (0.22 (0.12–0.47) vs. 0.18 (0.09–0.4), *p* = 0.053, Mann–Whitney U test), but ferritin (504 (264–1035) vs. 667 (386–1165.5), *p* = 0.018, Mann–Whitney U test) and IL-6 levels (60.9 (33–125) vs. 76 (40.5–139.65), *p* = 0.039, Mann–Whitney U test) were significantly lower in patients taking statin (Table 10).

Patients on statin therapy had significantly lower eGFR (60 (39–81) vs. 75 (52–90), *p* < 0.001, Mann–Whitney U test) and longer APTT (0.89 (0.81–1.01) vs. 0.86 (0.78–0.95), *p* = 0.006, Mann–Whitney U test) than patients not on statin therapy. Patients on statin therapy had significantly lower levels of cholesterol (4.2 (3.4–4.9) vs. 5.2 (4.4–6.2), *p* < 0.001, Mann–Whitney U test), triglycerides (1.6 (1.2–2.1) vs. 1.8 (1.4–2.4), *p* = 0.049, Mann–Whitney U test) and LDL (2.4 (1.8–3) vs. 3.1 (2.2–3.9), *p* < 0.001, Mann–Whitney U test). HDL was also significantly lower in patients on statin therapy (1.2 (0.96–1.3) vs. 1.3 (1.1–1.5), *p* = 0.002, Mann–Whitney U test) (Table 10).

There was no difference between the two groups in those requiring oxygen, high flow nasal cannula, corticosteroids, and remdesivir. Almost all patients in both groups received antibiotics. Patients on statin therapy were significantly less often on mechanical ventilation (27 [14%] vs. 52 [21%], *p* = 0.04, chi-square test).

In unadjusted analyses using the Mann–Whitney U test, patients receiving statin therapy had significantly lower IL-6 and ferritin levels compared with those not receiving statins (Table 10). Given the baseline differences in comorbidities between groups, multivariate linear regression analyses were performed using log-transformed IL-6 and ferritin levels as dependent variables. After adjustment for pre-specified baseline factors (age, coronary heart disease, chronic kidney disease, malignancy, and SpO_2_ at admission), the association between statin therapy and inflammatory markers was attenuated and was no longer statistically significant (Table 11).

## 4. Discussion

In our study of diabetic patients hospitalized with COVID-19, we found a high burden of cardiometabolic comorbidities and consistently elevated inflammatory markers, confirming that this population is at increased risk for severe clinical outcomes. In unadjusted analyses, patients on prehospital statin therapy had lower IL-6 and ferritin levels. However, these associations were attenuated and no longer statistically significant after adjustment for baseline comorbidities and disease severity. These findings suggest that the crude differences in inflammatory markers may be largely explained by baseline differences between groups. Interestingly, statin users required mechanical ventilation less often than non-users, pointing to a potential benefit in clinical outcomes despite the lack of an independent anti-inflammatory effect.

Emerging evidence suggests that statins, widely used for lipid-lowering therapy, exhibit significant immunomodulatory properties that may benefit diabetic patients infected with SARS-CoV-2. In individuals with diabetes mellitus chronic proinflammatory state contributes to worsened COVID-19 outcomes. Statins have been shown to reduce systemic inflammation, modulate immune responses, and improve endothelial function [22,24].

This study aimed to compare the clinical characteristics, outcomes, and laboratory parameters, including inflammatory markers, between patients with T2D who were on prehospital statin therapy and those who were not.

As was expected, the most elevated biomarkers in all subjects were CRP, ferritin and LDH. While statins have been reported to modulate inflammatory pathways, including IL-6 production via nuclear factor kappa B (NF-κB) inhibition, our results do not support an independent anti-inflammatory effect in this cohort of diabetic COVID-19 patients [25,26,27].

Supporting this, significantly lower mean and peak ferritin levels, regardless of diabetes status, were observed in COVID-19 patients receiving statins in a study conducted in Egypt [28]. Similarly, a study from New York found substantially reduced ferritin levels in both the overall cohort and among patients with diabetes who were treated with statins, while no such difference was observed in the non-diabetic subgroup [20].

Additionally, Zhang et al. reported that statin use during hospitalization was associated with significantly lower IL-6 and CRP levels, independent of diabetes status. These reductions were evident from the beginning of hospitalization and persisted throughout the clinical course, indicating a sustained anti-inflammatory effect of statins [29]. Similar findings were confirmed by Pereckaite et al., who observed significantly lower IL-6 levels in patients with both CVD and COVID-19, particularly in those receiving statins. The lowest IL-6 concentrations were found in this subgroup, suggesting a possible synergistic anti-inflammatory effect of statins and underlying cardiovascular comorbidity [30]. While the literature reports a potential anti-inflammatory benefit, in our study, the observed differences were likely influenced by underlying patient characteristics rather than a direct effect of statins.

In our study, we observed that diabetic COVID-19 patients on statin therapy exhibited significantly lower eGFR and prolonged activated partial thromboplastin time (APTT) compared to those not on statins.

Similar findings were reflected in a study conducted in New York, where serum creatinine levels were reported instead of calculated eGFR. Patients treated with statins had significantly higher creatinine concentrations compared to those not receiving statins (1.3 [0.92–2.4] vs. 1.1 [0.8–1.8]; *p* < 0.001), and this trend was also evident in diabetic patients on statin therapy (1.4 [1–2.8] vs. 1.3 [0.9–2.3]; *p* < 0.001) [20]. Estimated glomerular filtration rate in a study conducted in Northern Italy was slightly lower in patients receiving statin therapy (64 ± 23 mL/min) compared to those not on statins (67 ± 32 mL/min), but this difference was not statistically significant (*p* = 0.520) [31].

Several factors may contribute to the reduced eGFR in statin users. The reduced eGFR in statin users may reflect preexisting diabetic nephropathy, as these patients often have a more advanced cardiovascular and renal risk profile. Alternatively, the difference may be partially attributable to increased susceptibility to COVID-19-associated acute kidney injury (AKI) in a sicker subgroup, or, less commonly, statin-associated myopathy and rhabdomyolysis in the setting of critical illness [21,32,33].

The observed prolongation of APTT may be multifactorial. Although statins possess anti-inflammatory and antithrombotic properties that may influence coagulation and attenuate the hypercoagulable state seen in severe COVID-19, the use of anticoagulants such as low molecular weight heparin (LMWH), which does not affect APTT, was common among hospitalized patients. In our study, the difference in APTT between statin users and non-users was statistically significant but clinically minimal (0.89 [0.81–1.01] vs. 0.86 [0.78–0.95], *p* = 0.006), suggesting that factors other than anticoagulation intensity may contribute to the observed APTT variation. Statins mainly affect coagulation by reducing procoagulant factors and thrombin generation, without directly altering APTT, indicating that APTT changes in statin users likely reflect more complex mechanisms [34].

We also analyzed whether patients on statin therapy needed the same in-hospital therapy. There was no difference between the two groups in those requiring oxygen, high flow nasal cannula, corticosteroids and remdesivir. Notably, statin use was associated with a lower likelihood of requiring mechanical ventilation (13% vs. 21%, *p* = 0.032). This association remained significant after adjusting for key baseline factors in multivariate logistic regression (OR = 0.48, 95% CI 0.25–0.93, *p* = 0.03), suggesting an independent association with a lower likelihood of mechanical ventilation. These findings should be interpreted with caution, as intubation criteria were not standardized across clinicians and institutions.

This finding is consistent with previous research. A meta-analysis focusing on critically ill patients showed that prehospital statin use was associated with a lower likelihood of requiring mechanical ventilation [35]. Similarly, a preliminary study reported that COVID-19 patients on statins were less likely to require invasive mechanical ventilation compared to non-users. After adjusting for factors such as age, comorbidities, and baseline risk, statin therapy was associated with a more than 50% reduction in the risk of mechanical ventilation (adjusted odds ratio (aOR) = 0.45; 95% CI: 0.20–0.99; *p* = 0.048) [36].

A retrospective cohort study of hospitalized COVID-19 patients also demonstrated that in-hospital statin use was independently associated with a significantly reduced need for mechanical ventilation. After adjusting for comorbidities and other variables, patients treated with statins had a lower risk of requiring mechanical ventilation (adjusted hazard ratio (aHR) = 0.37; 95% CI: 0.26–0.53; *p* < 0.001). Similar results were observed in propensity score-matched analyses, where statin therapy remained associated with a lower risk (aHR = 0.51; 95% CI: 0.34–0.78; *p* = 0.002). Additionally, statin use was associated with reduced ICU admission rates (aHR = 0.69) and a lower risk of developing ARDS [29].

The study by Lohia et al. further supports these findings. Among all hospitalized COVID-19 patients, including a subgroup with diabetes, in-hospital statin therapy was associated with a lower likelihood of requiring mechanical ventilation. In the unadjusted analysis, statin use reduced the risk of mechanical ventilation by 56% (OR = 0.44; 95% CI: 0.28–0.70; *p* < 0.001), while in adjusted models, the association remained significant, showing a 61% risk reduction (OR = 0.39; 95% CI: 0.23–0.65; *p* < 0.001) [21]. Taken together, these findings support a potential beneficial role of statins in reducing ventilatory support needs, but should be interpreted cautiously in the context of non-standardized clinical endpoints.

While some studies have reported positive outcomes of statin use in COVID-19 patients, the evidence is not entirely consistent. For example, a large multicenter retrospective study did not find a significant association between prehospital statin use and reduced need for mechanical ventilation or mortality, even after adjusting for confounding factors using propensity score matching [37].

In contrast, a meta-analysis of 19 studies looking specifically at mechanical ventilation in COVID-19 patients found that statin use was linked to a significant reduction in risk (OR = 0.84; 95% CI: 0.78–0.92; *p* < 0.001) [38]. Overall, statins may help reduce the risk of severe respiratory complications in COVID-19, possibly due to their anti-inflammatory, endothelial-stabilizing, and immunomodulatory properties, but non-standardized ventilation criteria across studies warrant cautious interpretation [22].

Chronic glycemic control, usually measured by HbA1c, has been linked to worse COVID-19 outcomes in patients with diabetes, including a higher risk of severe disease and mortality. Some studies found that higher HbA1c levels were associated with these adverse outcomes [39,40], while others, such as the CORONADO study, did not show an independent effect after adjusting for other factors [41]. In our cohort, HbA1c was not systematically available, so we could not distinguish the impact of chronic glycemic control from acute hyperglycemia on inflammatory markers or clinical outcomes.

In patients with both T2D and COVID-19, we observed a combination of metabolic dysfunction, immune dysregulation, and systemic inflammation. In unadjusted analyses, prehospital statin use was associated with lower IL-6 and ferritin levels and with a lower frequency of mechanical ventilation. However, after adjustment for baseline comorbidities and disease severity, the differences in inflammatory markers were no longer statistically significant, indicating that they were largely driven by underlying patient characteristics. In contrast, the association between statin use and a lower likelihood of mechanical ventilation persisted. These findings reflect associations observed in our cohort and should not be interpreted as evidence of a causal protective effect.

Given the observational design and the possibility of residual confounding, our findings should be interpreted with caution. Prospective studies with standardized clinical endpoints are warranted to better define the potential role of statins in patients with T2D hospitalized with COVID-19.

## 5. Limitations of the Study

There are several limitations in our study. The exclusion criteria were relatively limited, which may have allowed for the inclusion of patients with confounding conditions that influenced inflammatory markers, such as autoimmune diseases, active malignancies undergoing chemotherapy, or those on chronic hemodialysis. Additionally, we used only four inflammatory biomarkers (CRP, PCT, ferritin, and IL-6). Including a broader range of cytokines and clustering them with sociodemographic and clinical variables could have allowed for the identification of distinct inflammatory phenotypes among patients with T2D and COVID-19. Analyzing intra- and intercluster patterns of inflammatory markers may also have provided deeper insight into the different mechanisms of chronic inflammation.

In addition, without data on diabetes duration and HbA1c, we were unable to assess how long-term glycemic control may have affected patient outcomes. Differences in inflammatory markers such as IL-6 or ferritin may therefore partly reflect chronic hyperglycemia rather than the effect of statin therapy alone [42]. The study population included only hospitalized patients, most of whom presented with more severe forms of COVID-19. Therefore, our findings may not be applicable to individuals with mild symptoms or those managed outside the hospital.

Although we performed multivariate adjustment for key baseline factors, the absence of propensity score matching and the lack of standardized criteria for initiating mechanical ventilation may have introduced residual confounding and limited the strength of causal inferences regarding clinical outcomes.

Due to its retrospective observational design, this study is inherently constrained in determining causality and may be influenced by unmeasured confounders. However, it was a so-called real-world research.

## Figures and Tables

**Table 1:** Basic characteristics of the patients (N = 440).

Variable	n/Median	%/Interquartile Range
Age (years)	70	(62–77)
Gender (female/%)	190	43.2%
Day of illness at admission	7	(4–10)
Duration of hospitalization (days)	11	(7–15)

**Table 2:** Clinical and anthropometric characteristics of the patients (N = 440).

Variable	Median	Interquartile Range
Body weight (kg)	88	(77–100)
Height (cm)	169	(162–175)
BMI (kg/m^2^)	30.8	(27.3–34)
Puls (/min)	89	(80–100)
SpO_2_ (%)	91	(85–95)
Systolic pressure (mmHg)	125	(110–140)
Diastolic pressure (mmHg)	77	(70–80)
Body temperature at admission (°C)	36.8	(36.4–37.1)

BMI—body mass index; SpO_2_—peripheral oxygen saturation; kg—kilograms; cm—centimeters; m—meters, /min—per minute, mmHg—millimeters of mercury; °C—degrees Celsius.

**Table 3:** Frequency of chronic disease (N = 440).

**Comorbidity**	**Number (%)**
Arterial hypertension	381 (87%)
Chronic kidney disease	136 (31%)
Coronary disease	107 (24%)
Heart failure	103 (23%)
Cerebrovascular disease	101 (23%)
Pulmonary disease	60 (14%)
Malignant disease	43 (10%)
Liver disease	24 (6%)

**Table 4:** Laboratory characteristics of the patients (N = 440).

Laboratory Parameter	Median	Interquartile Range
White blood cells (×10^9^/L)	7.1	(5.3–9.6)
Hemoglobin (g/L)	133	(119–144)
Thrombocytes (×10^9^/L)	210	(153–269)
CRP (mg/L)	110	(57.3–161.5)
PCT (µg/L)	0.2	(0.1–0.4)
Ferritin (µg/L)	571	(340–1105)
IL-6 (ng/L)	70	(36–133)
AST (U/L)	41	(30–63.8)
ALT (U/L)	31.5	(22–50)
eGFR (mL/min/1.73 m^2^)	69	(43–87)
D-dimer (µg/L FEU)	1193	(659–2333)
PT-INR	1	(0.9–1)
APTT	0.9	(0.8–1)
AT-3 (%)	1	(0.9–1.1)
Cholesterol (mmol/L)	4.6	(3.9–5.7)
Triglycerides (mmol/L)	1.7	(1.3–2.3)
HDL (mmol/L)	1.2	(1–1.4)
LDL (mmol/L)	2.7	(2.1–3.5)
Blood glucose (mmol/L)	11.4	(8.4–16.5)
LDH (U/L)	329.5	(251–441)

Abbreviations: CRP—C-reactive Protein, PCT—procalcitonin, IL-6—interleukin-6, AST—aspartate aminotransferase, ALT—alanine aminotransferase, eGFR—estimated glomerular filtration rate, PT-INR—prothrombin time—international normalized ratio, APTT—activated partial thromboplastin time, AT-3—antithrombin III, LDL—low-density lipoprotein, HDL—high-density lipoprotein, LDH—lactate dehydrogenase.

**Table 5:** Distribution of participants according to other regular therapies.

Medication	Number (%)
ACE-INH/ARB	303 (68.9)
Beta blocker	183 (41.6)
Calcium channel blockers	183 (41.6)
Diuretic	173 (39.3)
Acetylsalicylic acid	75 (17)
Oral anticoagulants	52 (11.8)

Abbreviations: ACE-INH/ARB—angiotensin-converting enzyme inhibitors/angiotensin receptor blockers.

**Table 6:** Distribution of participants according to antidiabetic therapy.

Antidiabetic Therapy	Number (%)
Insulin	123 (28)
Metformin	251 (57.2)
DPP-4i	117 (26.7)
Sulfonylurea preparations	105 (23.9)
SGLT-2i	27 (6.1)
GLP-1 RA	16 (3.6)
Pioglitazone	6 (1.4)

Abbreviations: DPP-4i—dipeptidyl peptidase 4 inhibitors, SGLT-2i—sodium glucose cotransporter 2 inhibitors, GLP-1 RA—glucagon-like 1 receptor agonist.

**Table 7:** Prehospital statin therapy and statin potency in the study population (N = 440).

Variable	Number (%)	Variable	Number (%)
Prescribed statin	197 (45)	Prescribed statin	197 (45)
Atorvastatin	148 (75)	Atorvastatin	148 (75)
Rosuvastatin	26 (13)	Rosuvastatin	26 (13)
Simvastatin	19 (10)	Simvastatin	19 (10)
Fluvastatin	4 (2)	Fluvastatin	4 (2)
Statin potency			
Low-potency	3 (1.5)		
Moderate-potency	133 (67.5)		
High-potency	61 (31)		

**Table 8:** Differences between the two groups according to chronic disease and hospital treatment: those who were treated with statin therapy prehospitally, and those who were not (N = 440).

Variable	On Statin (197)	Not on Statin (243)	*p* Value *
Arterial hypertension	186 (94%)	195 (80%)	**<0.001**
Coronary disease	75 (38%)	32 (13%)	**<0.001**
Heart failure	58 (29%)	45 (19%)	**0.009**
Pulmonary disease	34 (17%)	26 (11%)	0.051
Liver disease	6 (3%)	18 (7%)	0.57
Chronic kidney disease	82 (42%)	54 (22%)	**<0.001**
Cerebrovascular disease	65 (33%)	36 (15%)	**<0.001**
Malignant disease	18 (9%)	25 (10%)	0.61
Hospital therapy			
Oxygen therapy	143 (73%)	180 (74%)	0.746
High flow nasal cannula	27 (14%)	43 (18%)	0.26
Mechanical ventilation	27 (14%)	52 (21%)	**0.04**
Corticosteroids	162 (82%)	204 (84%)	0.63
Remdesivir	42 (21%)	69 (28%)	0.097
Antibiotic	192 (98%)	233 (96%)	0.37

* Chi-square test. Bold values denote statistical significance.

**Table 9:** Multivariate logistic regression analysis for mechanical ventilation.

* Mechanical Ventilation	β	*p* Value	OR	95% CI
Age	−0.008	0.60	0.99	0.96–1.02
Coronary artery disease	0.336	0.35	1.40	0.69–2.83
Chronic kidney disease	0.181	0.60	1.20	0.61–2.37
Malignancy	0.673	0.13	1.96	0.82–4.67
SpO_2_ at admission	−0.107	**<0.001**	0.90	0.87–0.93
Blood glucose	0.026	0.18	1.03	0.99–1.07
Statin therapy	−0.733	**0.03**	0.48	0.25–0.93

* Multivariate logistic regression analysis (enter method) adjusted for age, coronary artery disease, chronic kidney disease, malignancy, SpO_2_ at admission, and blood glucose level. Abbreviation: OR—odds ratio; CI—confidence interval. Bold values denote statistical significance.

**Table 10:** Differences in clinical characteristics between the two groups: those who were treated with statin therapy prehospitally, and those who were not (N = 440).

Variable	On Statin (n = 197)	Not on Statin (n = 243)	*p*
Age, years	70 (64–77)	70 (62–78)	0.635
Length of stay, days	10 (6–15)	10 (7–15)	0.737
Heart rate, bpm	88 (77–99)	90 (80–102)	**0.029**
SpO_2_, %	91 (85–94)	90 (83–94)	0.184
Body temperature, °C	36.7 (36.4–37.2)	36.8 (36.4–37.1)	0.648
White blood cells (×10^9^/L)	6.9 (5.3–9.1)	7.4 (5.1–9.9)	0.283
Hemoglobin (g/L)	129 (116–143)	134 (122–146)	**0.007**
Trombocytes (×10^9^/L)	208 (147–274)	211 (160–269)	0.464
CRP (mg/L)	99 (52–158)	133 (66–166)	0.208
PCT (µg/L)	0.2 (0.1–0.5)	0.2 (0.1–0.4)	0.053
Ferritin (µg/L)	504 (264–1035)	667 (386–1165.5)	**0.018**
IL-6 (ng/L)	60.9 (33–125)	76 (40.5–139.7)	**0.039**
AST (U/L)	45 (31–65)	39 (29–63)	0.22
ALT (U/L)	31 (22–50)	33 (21–50)	0.956
eGFR (mL/min/1.73 m^2^)	60 (39–81)	75 (52–90)	**<0.001**
D-dimer (µg/L)	1161.5 (676.3–2117.8)	1206 (647.5–2520.8)	0.904
PT-INR	1 (0.9–1.1)	1 (0.9–1)	0.413
APTT	0.9 (0.8–1)	0.9 (0.8–1)	**0.006**
AT-3 (%)	1 (0.9–1.1)	1 (0.9–1.1)	0.651
Cholesterol (mmol/L)	4.2 (3.4–4.9)	5.2 (4.4–6.2)	**<0.001**
Triglycerides (mmol/L)	1.6 (1.2–2.1)	1.8 (1.4–2.4)	**0.049**
HDL (mmol/L)	1.2 (0.96–1.3)	1.3 (1.1–1.5)	**0.002**
LDL (mmol/L)	2.4 (1.8–3)	3.1 (2.2–3.9)	**<0.001**
Blood glucose (mmol/L)	10.9 (8.1–16.1)	11.9 (8.9–17.5)	0.125
LDH (U/L)	314 (244.1–415.8)	337.5 (255–454.3)	0.138

Independent-Samples Mann–Whitney U test. Abbreviations: bpm—beats per minute, SpO_2_—peripheral capillary oxygen saturation, CRP—C-reactive protein, PCT—procalcitonin, IL-6—interleukin-6, AST—aspartate aminotransferase, ALT—alanine aminotransferase, eGFR—estimated glomerular filtration rate, PT-INR—prothrombin time—international normalized ratio, APTT—activated partial thromboplastin time, AT-3—antithrombin III, HDL—high-density lipoprotein, LDL—low-density lipoprotein, LDH—lactate dehydrogenase. Bold values denote statistical significance.

**Table 11:** Multivariate linear regression analysis for log-transformed inflammatory markers.

	log(IL-6) (95% CI)	*p* Value	log(Ferritin) (95% CI)	*p* Value
Age	0.004 (0–0.009)	0.08	−0.009 (−0.014–0.005)	<0.001
Coronary artery disease	0.035 (−0.085–0.156)	0.56	0.063 (−0.043–0.169)	0.25
Chronic kidney disease	0.101 (−0.018–0.221)	0.10	0.085 (−0.022–0.191)	0.12
Malignancy	0.044 (−0.143–0.231)	0.64	0.045 (−0.109–0.2)	0.56
SpO_2_ at admission	−0.009 (−0.015–0.003)	0.002	−0.006 (−0.011–0)	0.03
Statin therapy	−0.087 (−0.194–0.019)	0.11	−0.090 (−0.184–0.004)	**0.06**

Dependent variables: log-transformed IL-6 and log-transformed ferritin. Models adjusted for age, coronary artery disease, chronic kidney disease, malignancy, and SpO_2_ at admission. Bold values denote statistical significance.

## Data Availability

The raw data supporting the conclusions of this article will be made available by the authors on request due to privacy concerns.
